# Effects of Exercise Speed and Circle Diameter on Markers of Bone and Joint Health in Juvenile Sheep as an Equine Model

**DOI:** 10.3390/ani15030414

**Published:** 2025-02-02

**Authors:** Renee M. Harbowy, Brian D. Nielsen, Aimee C. Colbath, Cara I. Robison, Daniel D. Buskirk, Alyssa A. Logan

**Affiliations:** 1 Department of Animal Science, Michigan State University, 474 S. Shaw Lane, East Lansing, MI 48824, USA; bdn@msu.edu (B.D.N.); oconn107@msu.edu (C.I.R.); buskirk@msu.edu (D.D.B.); 2 Department of Clinical Sciences, Cornell University College of Veterinary Medicine, 930 Campus Road, Box 30, Ithaca, NY 14853, USA; ac2399@cornell.edu; 3 School of Agriculture, Middle Tennessee State University, 314 W. Thompson Ln., Murfreesboro, TN 37129, USA; alyssa.logan@mtsu.edu

**Keywords:** circular exercise, lunging, cartilage, musculoskeletal, exercise adaptations, horse, osteoarthritis, walker

## Abstract

Though circular exercise is commonly used in equestrian disciplines, it may be at the detriment of horses’ musculoskeletal system. To investigate the effects of circular exercise on bone and joint health, 42 lambs were randomly assigned to a non-exercised control, straight-line, small circle, or large circle exercise regime at a slow (1.3 m/s) or fast (2.0 m/s) speed for 12 wk. Blood samples were taken biweekly. Animals were humanely euthanized upon study completion, and the fused third and fourth metacarpals were collected for biomechanical testing and bone density analysis. Fast groups were found to have more bone formation and less resorption activity than slow groups as evidenced by serum biomarker concentrations (*p* < 0.05). Sheep in the large fast group tended to have greater flexural rigidity and fracture force for the outside leg compared to the inside leg (*p* < 0.1). Sheep in the small slow group tended to have increased bone mineral density of the outside leg compared to the inside leg, whereas the opposite occurred in the large slow group (*p* < 0.1). These results provide further evidence for potential asymmetric musculoskeletal adaptations to circular exercise while emphasizing the importance of speed as a positive influence on bone metabolism and strength.

## Simple Summary

Circular exercise is a common practice in many disciplines, whether it be in the form of lunging, a mechanical walker, or ridden exercise. However, how horses adapt to circular exercise may put their bone and joint health at risk. To evaluate how the musculoskeletal system responds to circular exercise, juvenile sheep were used as a model for young horses. Circular exercise was found to influence traits of bone quality, including density and fracture force. Speed and circle diameter also influenced markers of bone formation and bone resorption. Though no joint damage was found, it is possible that joint changes were undetected and tighter or faster circles would have produced different results. It is important for one to consider factors such as speed and circle diameter in their exercise programs to help ensure the musculoskeletal health of equine athletes.

## 1. Introduction

Preventing musculoskeletal injuries in horses is a major concern for the equine industry as these injuries are the leading cause for horse loss across many disciplines [1]. Even in a mild case of injury, lameness is the leading cause of lost training days [1]. Thus, this is not only a glaring welfare concern for equine athletes but is a major economic burden for owners and trainers. Further, damage to the musculoskeletal system can lead to long-term debilitating conditions such as osteoarthritis (OA). Osteoarthritis is one of the most frequently reported chronic conditions in horses and accounts for up to 60% of all lameness cases [2,3,4,5]. This is often considered a common consequence following many equine athletes’ careers and may be due to several influencing factors, with one such factor being abnormal loading of the joint [6,7]. Thus, overloading the joint and compromising supporting joint structures are both concerns in maintaining musculoskeletal integrity.

Sprint exercise is well known to have positive effects on bone metabolism, and training young animals at speed may reduce the risk of injury by promoting denser, stronger bones [8,9,10]. Similarly, exercise is beneficial for cartilage maturation and regulation of joint homeostasis, though excessive or abnormal loading has been associated with the development of joint disease [2]. However, horse owners and trainers tend to utilize longer, slower exercise sessions—often performed in a circle—rather than straight-line sprints. For instance, circular exercise may occur in the form of lunging or placing horses on a mechanical walker, often for the purpose of improving fitness, allowing release of pent-up energy, or as a perceived “safe” alternative to turnout [11,12,13]. Circular exercise also occurs frequently during ridden exercise, both during competition and training. Specifically, disciplines such as reining, barrel racing, or show jumping often include very small circles or tight turns performed at relatively quick speeds. Though exercising on a curve or circle is used frequently during training or competition, it may be at the detriment of the horse’s musculoskeletal system.

As horses exercise on a curve, they increase their lean angle toward the center of the turn to aid in offsetting centripetal force and maintaining balance, with a greater angle associated with smaller turns and faster speeds [14]. As the horse abducts the limb from the body, the strain environment of the outside leg is increased—thus increasing the strain placed on the bone, peak vertical ground reactions forces, and hoof loading area [14,15,16,17]. Further, the inside limb may experience increased duty factor, greater limb inclination, and increased torsion [14,18]. Thus, circular exercise inherently results in asymmetric limb loading.

For instance, amongst thoroughbred racehorses, it has been documented that the risk of fracture to the outside forelimb was the greatest on tracks with the smallest turn radii [19]. Similarly, underbanked tracks for standardbred racing have demonstrated increased strain on the outside limbs, with proper banking resulting in angle relief and decreased risk of injuries [20]. In a recent study using calves as a model for horses, circular exercise resulted in changes in increased dorsopalmar diameter of the fused third and fourth metacarpal (MCIII and IV) on the inside leg and increased glycosaminoglycan (GAG) on the outside leg [21]. Another earlier study, which used sheep as a model species, found greater macroscopic evidence of cartilage damage in circularly exercised animals [22]. Thus, concern is warranted for how this style of exercise influences the musculoskeletal system and risk for equine injury.

Measuring changes in bone and cartilage are necessary to truly determine the effects of exercise. Serum biomarkers such as osteocalcin (OCN) and crosslinked C-telopeptides of type I collagen (CTX-I) are useful in determining relative bone formation and resorption activity, respectively [23]. Bone can further be assessed through width and mineral density measurements via computed tomography (CT) scans, which allows for greater insight regarding structural changes of bone [24]. Biomechanical testing of bone allows for measurements of the bone’s ultimate strength, indicating how resistant it is to fractures [25]. Together, these factors create a comprehensive image of bone quality for researchers. Similarly, a number of factors can be analyzed to investigate effects of exercise on the joint environment. Synovial fluid prostaglandin E_2_ (PGE_2_) concentrations are often used as a marker of inflammation within the joint and have been found to be elevated in horses with OA or synovitis [26,27]. Cartilage DNA also provides insight into joint health as a relative marker of cellularity; damage to joints has been found to result in decreased cellularity, and subsequently, DNA content [28]. Similarly, cartilage GAG content is a useful marker of extracellular matrix (ECM) quality. In damaged cartilage, such as with OA or injury, GAG content has been noted to decrease [29].

Though many studies have investigated the effects of circular exercise on gait kinematics and kinetics, few have delved into the effects this exercise has on bone and joint quality. Thus, the objective of this study was to determine the influence of circle diameter and speed on markers of bone and joint quality in the distal forelimb. To achieve this, lambs were used as a model for young horses, which have previously been used as a successful model for musculoskeletal research [22,30]. It was hypothesized that smaller circle diameter and faster speeds would result in more asymmetrical changes to bone and negative effects on joint health.

## 2. Materials and Methods

### 2.1. Animals and Housing

**Figure 1:**
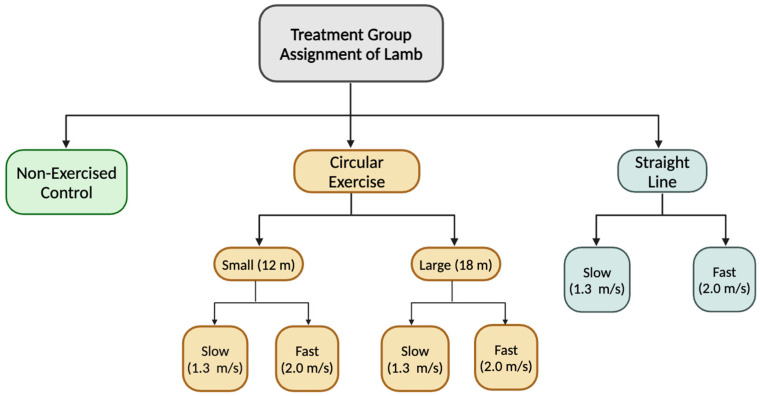
Treatment groups for animals based on exercise style (straight line, large circle, small circle, or no exercise) and speed (slow (1.3 m/s) or fast (2.0 m/s)).

All use of animals was approved by the Michigan State University Institute of Animal Care and Use Committee (IACUC PROTO202200234; approved 16 September 2022). Forty-two lambs (21 ewes and 21 wethers) aged 4 ± 1 mo with an average weight of 40 ± 1 kg were obtained from the Michigan State University Sheep Teaching and Research Center. On d 0, lambs were measured for weight and height then striated into one of seven treatment groups. Each treatment group was composed of 6 animals: 3 ewes and 3 wethers. Exercise was conducted 4 d/wk for 12 wk on a mechanical walker (circular exercise) with a diameter of 12 m (small) or 18 m (large) or a treadmill (straight line) at a speed of 1.3 m/s (slow) or 2.0 m/s (fast) with one group serving as a non-exercised control (Figure 1). The footing in the walker was composed of approximately 5 cm of 2NS sand on a crushed asphalt base. Circularly exercised animals tracked right (clockwise) to ensure the inside and outside limbs remained consistent. With this, for the circularly exercised groups, the left leg was always the outside leg, and the right leg was always the inside leg. Further, clockwise was chosen as the exercise direction for ease of loading sheep into the walker as the design of the entryway encouraged them to track right. Animals started exercising at 390 m/d, which increased by 390 m weekly until they reached 2340 m/d (Table 1). When not exercising, animals were housed in two straw-bedded pens with areas of 35 m^2^ and 33 m^2^ that allowed for ad libitum access to clean water and hay. Animals were fed a grain–mineral mix once daily at a rate of 0.65 kg/hd/d.

### 2.2. Sample Collection

A chute system was assembled near the housing pens, which was used to sort animals into their exercise groups, as well as perform blood draws every two weeks beginning on d0. Blood samples were collected on non-exercise days via jugular venipuncture into 10 mL serum vacutainer tubes (Evacuated BD Vacutainer Serum Tubes, Becton, Dickson, and Company, Franklin Lakes, NJ, USA). Blood samples were centrifuged after being allowed to clot for 2 h, and serum was aliquoted and frozen immediately to be stored at −20 °C. On d83 to d85, all animals were humanely euthanized at the Michigan State University Meat Laboratory. The distal forelimb was removed from the animal at the mid-radius. Synovial fluid (SF) was collected from the carpus of each leg primarily from the radiocarpal, but composite samples were accepted if SF was also collected from the intercarpal or carpometacarpal joints due to limited fluid volume. Once SF was collected, it was immediately placed onto dry ice and then stored at −80 °C until analysis. Each joint was then opened to expose the joint surface, and a cartilage sample was taken from the carpometacarpal joint with a scalpel. Cartilage was also placed on dry ice at time of collection, and then stored at −20 °C until analysis. After the samples were collected, legs were labeled and placed in a temporary chiller (4.8 °C) overnight until they were computed tomography scanned. After computed tomography scans were completed, legs were stored in a freezer (−20 °C) until biomechanical testing of the fused third and fourth metacarpal (MCIII and IV) was completed.

### 2.3. Computed Tomography Scans

Computed tomography (CT) scans were completed at the Michigan State University College of Veterinary Medicine using a GE Revolution Evo Scanner (Boston, MA, USA). The right and left distal forelimbs of each animal were scanned within 36 h of collection. The position was set to a head-first supine position. Slice density was specified to 0.625 mm with settings of 120 kV and 320 mA. Each scan had a 100 mm field of view with a 512 × 512 matrix size, resulting in a 0.195 mm × 0.195 mm pixel and a subsequent voxel volume of 0.024 mm^3^. A calcium hydroxyapatite phantom was used in each of the scans to provide a reference for bone mineral density (BMD) with rows of 0, 75, and 100 mineral/cm^3^. The average HU was recorded at 10 locations within each mineral density section of the phantom. These HU values were then graphed against the known concentration of minerals to create a regression line. The equation of this line was used to convert HU into mg mineral/cm^3^ to determine BMD. This method for calculating BMD has been previously used and reported [21].

All CT scans were analyzed using Mimics 24.0 (Materialise, Leuven, Belgium) with measurements taken at the midpoint of the MCIII and IV. The midpoint was determined by measuring the total length of each bone. Bone mineral density, cross-sectional area, cortical area, dorsopalmar diameter, mediolateral diameter, and cortical widths were all recorded. Three scans were found to have significant artifacts present that compromised the ability to measure BMD. Due to these bones already having undergone biomechanical testing, they could not be rescanned and were removed from the dataset.

### 2.4. Biomechanical Testing

All legs were moved from the freezer (−20 °C) to thaw for 12 h at 4.8 °C to excise the MCIII and IV and remove all soft tissue from the bone using a scalpel. Once cleaned, the MCIII and IV was measured for length as well as dorsopalmar and mediolateral diameters at 25%, 50% and 75% of the total bone length. After measurements were taken, bones were each wrapped in a paper towel saturated with saline and replaced in freezer storage (−20 °C) until testing. Bones were removed from the freezer and allowed to thaw at 4.8 °C three days before testing. On the day of testing, bones were removed from refrigeration and allowed to warm to room temperature. Biomechanical testing of the MCIII and IV was performed at the Michigan State University Department of Plant Biology using a 3-point bending test using an electromechanical universal testing machine (Model 4202, Instron Corp., Canton, MA, USA) equipped with a 10 kN load cell. Right and left MCIII and IV were placed individually with the palmar aspect of the bone facing upwards towards the force applicator and the dorsal aspect of the bone under tension. Bottom supports were placed 80 mm apart, with the upper force applicator applied centrally between the two bottom supports (40 mm from each). Samples were loaded until failure at a rate of 10 mm/min with a data acquisition rate of 100 Hz. Fracture force was determined to be the maximum amount of force applied before mechanical failure of the bone.

Bones were modeled as a hollow ellipse and all calculations were performed in accordance with guidelines from American Society of Agricultural and Biological Engineers and previous methodology of this laboratory [8,21,31,32]. Dimensions of bone necessary for calculations of moment of inertia, Young’s modulus, and flexural rigidity were acquired from CT scan analysis using Mimics software. The moment of inertia (*MOI*, mm^4^) was calculated as follows:

$$MOI=0.049[\left(B\times D^{3}\right)-\left(b\times d^{3}\right)]$$

where *B* is the outer lateromedial diameter of the bone, *D* is the outer dorsopalmar diameter, *b* is the inner lateromedial diameter, and *d* is the inner dorsopalmar diameter, and 0.0049 is a constant. Young’s modulus (*E*, N/mm^2^) was calculated as follows:

$$E=\left(\frac{F}{d}\right)\left(\frac{L^{3}}{48(MOI)}\right)$$

where *F* is the applied force, *d* is the displacement of the bone, *L* is the length between lower posts, and 48 is a constant. Flexural rigidity (EI, N·mm^2^) was calculated as the product of the moment of inertia and Young’s modulus.

### 2.5. Osteocalcin

Serum samples from the sheep were analyzed for OCN concentration as a marker osteoblast activity and thus bone formation. Assays were performed using the commercially produced MicroVue Bone Osteocalcin Enzyme Immunoassay kit (Quidel, San Diego, CA, USA). All samples were diluted 1:8 with wash buffer, and analysis was performed following kit instructions. Results with a coefficient of variation of 10% or lower were accepted.

### 2.6. Crosslinked C-Telopeptides of Type-I Collagen

Sheep serum samples were evaluated for concentrations of CTX-1 as a marker of osteoclast activity and thus bone resorption. Assays were performed using the commercially produced Serum Crosslaps kit by Immunodiagnostics Systems (Gaithersburg, MD, USA). All samples were run neat, and analysis was performed following kit instructions. Results with a coefficient of variation of 10% or lower were accepted.

### 2.7. Ratio of Osteocalcin to Crosslinked C-Telopeptides of Type-I Collagen

A ratio of OCN to CTX-1 was calculated to gain an understanding of the relative relationship of bone formation and bone resorption. Values for the ratios were calculated as the OCN level divided by the CTX-1 level, both measured in ng/mL.

### 2.8. Prostaglandin E_2_

Synovial fluid samples were analyzed for PGE_2_ concentration as a marker of inflammation within the joint. Assays were performed using a commercially produced PGE_2_ ELISA kit (Enzo, Farmingdale, NY, USA). Samples were first digested with a 50 μg/mL hyaluronidase solution with hyaluronidase sourced from bovine testes. After digestion, samples were diluted 1:2 with assay buffer. Due to some samples falling outside of the sensitivity range, select individual samples were rerun neat. Analysis was performed according to kit instructions. Coefficients of variation at or below 15% were accepted.

### 2.9. Glycosaminoglycan

Cartilage samples were digested with papain for determination of GAG concentrations as a measure of ECM quality. To achieve this, a dimethylmethylene blue assay was used. This procedure has been previously validated and has been used in the methodology of previous investigations by this laboratory [21,33]. The cationic 1,9-dimethylene blue dye binds to anionic GAG, resulting in a colorimetric assay. A chondroitin sulfate standard was used for generation of a linear curve against which sample values were determined. Digested samples were diluted 1:25 with dilution buffer. Coefficients of variation at or below 10% were accepted.

### 2.10. DNA

Digested cartilage samples were also analyzed for DNA content as a measure of cellularity. Assays were performed using the commercially produced Qubit dsDNA High Sensitivity kit (ThermoFisher Scientific, Waltham, MA, USA). In total, 15 µL of digested sample was added to 185 µL of working solution for a total tube volume of 200 µL according to kit instructions. The Qubit 2.0 Fluorometer was calibrated using the provided standards to ensure accurate reading of the samples.

### 2.11. Statistical Analysis

All statistical analyses were performed in SAS 9.4 (SAS Institute Inc., Cary, NC, USA). OCN and CTX-1 were run using Proc Glimmix with the main effects of day, treatment, and interaction of d by treatment. The ratio of OCN to CTX-1 was also analyzed using Proc Glimmix with the main effects of day, treatment, and interaction of day by treatment. Data from CT scans, biomechanical testing, and PGE_2_ concentrations were run using Proc Mixed with the fixed effects of sheep, treatment, leg and interaction of treatment by leg. The GAG and DNA concentration data were grouped by leg and analyzed with the fixed effects of sheep and treatment. All data are reported as the least squares mean (LSMean) ± the standard error of the mean (SEM). Significance was set at *p* ≤ 0.05 and trends are discussed at *p* ≤ 0.1.

## 3. Results

### 3.1. Computed Tomography Scans

A significant treatment by leg interaction was found for the dorsal cortex of the fused MCIII and IV (*p* = 0.033), with the small slow treatment group tending to have a higher dorsal cortex density for the outside leg compared to the inside leg (*p* = 0.078) and the straight fast group having greater density for the left leg compared to the right (*p* = 0.008) Additionally, there was a trend for a treatment by leg interaction for the medial cortex density (*p* = 0.074). This appears to be driven by a significant effect of treatment by leg for the large slow and straight fast exercise groups (*p* = 0.018 and *p* = 0.019, respectively), with the large slow treatment group having a lower lateral cortex density for the outside leg compared to the inside and the straight fast group having greater BMD for the left leg than the right leg (Table 2). There was no effect of treatment, leg, or the interaction of treatment by leg on lateral or palmar cortex density. There was no effect of treatment, leg, or the interaction of treatment by leg for midpoint whole slice density (Table 2).

There was no effect of treatment or leg on bone length for the MCIII and IV. Similarly, there was no effect of treatment or leg for dorsal, lateral, medial, or palmar cortical widths nor whole slice, medullary mediolateral, or medullary dorsopalmar diameters. The interaction between treatment group and leg was insignificant for all measures of bone length and width. There was no effect of treatment, leg, or the interaction of treatment by leg for the cortical area or cross-sectional area.

### 3.2. Biomechanical Testing of the Fused Third and Fourth Metacarpal

Moment of inertia (*MOI*) was not significantly affected by treatment, leg, or the interaction of treatment by leg. Similarly, Young’s modulus or the modulus of elasticity (E) showed no significant effect of treatment, leg, or the interaction of treatment by leg. There was no treatment effect nor leg effect on flexural rigidity (EI), though there was a trend for a treatment by leg effect (*p* = 0.08) with the large fast exercise group tending to have greater EI for the outside leg compared to the inside leg. Similarly, there was no treatment or leg effect on fracture force (ultimate strength), though there was a trend for a treatment by leg effect (*p* = 0.08) with the large fast exercise group tending to have greater ultimate strength for the outside leg compared to the inside leg (Table 3).

### 3.3. Serum Osteocalcin

There was a significant effect of treatment (*p* = 0.002) and day (*p* < 0.001) on serum OCN concentrations. The small fast and straight fast treatment groups had greater OCN concentrations than the large fast, large slow, small slow, and straight slow treatment groups. Serum OCN was the greatest on d0 (*p* < 0.001). Concentrations of OCN decreased by d14 (*p* < 0.001) and remained the same until d28 (*p* = 0.16). The concentration of OCN then decreased again by d42 (*p* = 0.01) and stayed the same for the remainder of the study, except for a small increase on d70 (*p* = 0.04) relative to d42. The interaction between day and treatment was insignificant (Table 4).

### 3.4. Serum Crosslinked C-Telopeptides of Type-I Collagen

There was a significant effect of treatment (*p* < 0.001) and day (*p* < 0.001) on serum CTX-1 concentrations. The large slow and small slow treatment groups had greater CTX-1 concentrations when compared to the control, large fast, small fast, and straight fast, and straight slow treatment groups. The straight slow treatment group also showed greater concentrations in comparison to the straight fast group. Serum CTX-1 concentrations remained the same from d0 to d28, with a slight increase on d42. These levels then returned similar to the baseline, followed by another increase on d82 at which concentrations reached their peak. The interaction between day and treatment group was insignificant (Table 5).

### 3.5. Serum Ratio of OCN to CTX-1

The ratio of OCN to CTX-1 had a significant effect of treatment (*p* < 0.001) and day (*p* < 0.001), but the interaction of day by treatment was insignificant (Table 6). Control, large fast, small fast, and straight fast exercise groups all showed greater ratios than the large slow and small slow exercise groups. Aside from the control treatment, values were also increased in comparison to the straight slow treatment group. The straight fast treatment group also showed greater values compared to the control treatment group. Day 0 had a greater ratio of OCN to CTX-1 than all other days. The ratio between concentrations remained the same from d14 to d28, then decreased again by d42, and then increased further to d56. From d56 to d70, the ratio remained the same before a final decrease on d82.

### 3.6. Joint Health Biomarkers

There was no effect of treatment, leg, or interaction of treatment by leg for SF PGE_2_ concentrations. For cartilage DNA and GAG concentrations, samples were grouped by leg (left or right). There was no effect of treatment on cartilage DNA or GAG concentrations for right or left legs.

## 4. Discussion

Using lambs as a model for young horses to observe changes that occur to the musculoskeletal system proved to be useful. The hypothesis that smaller circle diameter and greater exercise speeds would result in greater indication of bone alteration and joint damage was able to be partially accepted. Results from this study showed that the musculoskeletal system does adapt differently to various exercise styles, marked by differences found in OCN and CTX-1 concentrations, bone mineral density, and fracture force. However, no measurable influence on joint health parameters was detected in this study.

Serum OCN concentrations, a marker of bone formation, revealed a significant effect of treatment with the straight fast and small fast treatment groups having greater concentrations in comparison to the straight slow, large slow, small slow, and large fast treatment groups (Table 4). These results indicate that faster exercise groups generally experienced increased bone formation due to increased strain associated with greater speed. This finding supports decades of work establishing that increasing strain has positive effects on bone formation activity [8,9,10,34,35,36,37,38]. Further, the increased serum concentrations of OCN for the small fast and straight fast treatment groups in this study, which were still relatively slow at 2.0 m/s, suggest that even small increases in speed may promote bone formation activity.

In a complementary fashion, a significant effect of treatment was also found for serum CTX-1 concentrations—a marker of bone resorption—with large slow and small slow exercise groups having increased values compared to the control, large fast, small fast, straight fast, and straight slow treatment groups (Table 5). The straight slow treatment group also had greater concentrations when compared to the straight fast treatment group. These data indicate that slow treatment groups had generally greater resorptive activity than fast treatment groups, supporting previous work establishing that slow or low-intensity exercise does not promote increased bone formation [39]. Further, it has been shown that serum CTX-1 concentrations were higher in stalled versus pastured horses [40]. As such, these results of the present study not only agree with previous work but also provide evidence that low-intensity exercise may promote resorptive activity of bone.

A comparison of ratios of OCN to CTX-1 further exemplified the patterns seen with OCN and CTX-1 concentrations individually (Table 6). Fast exercise groups had greater serum ratios of OCN to CTX-1 than slow exercise groups, indicating that faster speeds result in less resorption and more formation, or vice versa, that slower speeds result in more resorption and less formation.

The straight slow treatment group demonstrating lower CTX-1 concentrations, compared to the large slow and small slow treatment groups, may be due to differences in footing between sheep exercised using the mechanical walker versus the treadmill. The belt of the treadmill used to conduct straight-line exercise was a harder surface in comparison to the sand-based mechanical walker used for circular exercise, likely resulting in greater concussion and, in turn, bone strain for the straight-line sheep compared to their circularly exercised speed counterparts. Similarly, this footing difference likely played a role in the straight fast exercise group having greater OCN concentrations compared to the large fast exercise group.

Control lambs having serum OCN and CTX-1 concentrations similar to exercised lambs is likely due to the sheep engaging in free exercise (play) with their penmates, despite being housed confined pen areas (33–35 m^2^). Similar results have been found in horses previously, where pasture-reared horses engaged in enough free exercise to have greater lateral MCIII bone density compared to their stalled counterparts despite fewer forced exercise sessions [41]. Similarly, non-exercised horses on pasture have been shown to have bone density similar to endurance-trained horses kept in stalls [39].

The fact that the large fast treatment group was more similar to the slow treatment groups aligns with the hypothesis of this study. Smaller circle diameters are suspected to result in greater musculoskeletal adaptations compared to larger circle diameters due to increased lean angles [14,15,16,17]. Therefore, these results suggest that large circles, even at a faster speed, may result in loading comparable to small circles at slower speeds, which is likely due to similar lean angles. Similarly, exercising on a large circle diameter was expected to be more similar to straight-line exercise due to more normal loading, which is supported by OCN and CTX-1 concentrations being the same between the large fast and straight slow exercise groups. Only in the evaluation of the ratio of OCN to CTX-1 concentrations was the large fast treatment found to be different from the slow treatment groups, indicating that there may have been a slight favor of the bone toward less resorptive activity due to the greater speed that was not detected by evaluation of CTX-1 or OCN alone.

Serum OCN concentrations also showed a significant effect of day with concentrations generally decreasing over time, though they remained consistent from d14 to d28 and demonstrated a small increase on d70 (Table 4). These changes in OCN concentrations likely represent acclimation to exercise early in the study period and slowing of bone growth as animals matured. These patterns of OCN associated with training acclimation and maturation have been noted in previous studies [21,42,43]. While increased OCN levels on d70 were not expected, they may be attributable to the onset of cooler weather. This study was conducted from September to December in Michigan, USA. Thus, serum samples on d70 were collected in late November, around the time winter-like temperatures start to set in, with an average daily high temperature of 3.6 °C in the week leading up to d70. For comparison, the average daily high temperature was 17.6 °C in the week leading up to d56. Cooler weather may have led to animals engaging in more free exercise (play) due to decreased thermal loads as well as an increased willingness to engage in forced exercise sessions. Thus, all sheep may have experienced greater loading associated with more intensive free- and forced-exercised sessions, stimulating an increase in bone formation. Free exercise influencing bone formation activity has been documented previously in pasture-raised horses [39,41].

Serum CTX-1 concentrations also showed a significant effect of day (Table 5). Concentrations remained the same from d0 to d28, with a slight increase on d42 compared to d14 and d28. Concentrations then decreased again on d56 and were similar to the first four weeks of exercise until another increase on d82 in which they surpassed baseline values. Interestingly, this pattern appears to be the opposite of that observed with OCN, and similar findings have been documented regarding increased CTX-1 concentrations after acclimation of exercise and with maturation [21,44]. The serum ratio of OCN to CTX-1 concentrations was also found to decrease over time, demonstrating how both acclimation to an exercise regimen and age influence the dynamics of bone metabolism through the balance of formation and resorption activity.

Computed tomography scans revealed no differences in bone length or cross-sectional area indicating that the sheep had relatively uniform conformation and growth. No treatment differences were found for BMD within each cortex or whole-slice analysis, nor were treatment differences detected for fracture force, *MOI*, E, or EI. However, the large fast exercise group showed a trend for the outside leg to have greater flexural rigidity (EI) and fracture force in comparison to the inside leg (Table 3). Similarly, sheep in the small slow exercise group tended to have increased BMD for the medial aspect of the outside leg compared to the inside leg (Table 2). In contrast, sheep in the large slow treatment group had decreased medial BMD of the outside leg compared to the inside leg. These results do appear to be a logical result when considering the forces of circular exercise as the outside leg is found to experience increased strain as lean angle increases [14,15,16], which should result in increased bone density. Thus, these trends indicate that differences in circle diameter, even at the same speed, may result in asymmetric changes between inside and outside legs with smaller circles promoting increased bone density in the outside leg, whereas large circles, at the same speed, did not. However, the tendency for increased EI and fracture force in the outside leg of the large fast exercise groups suggests that loading differences between legs occur when exercising in a large circle once speed is increased. Though the straight fast exercise group also showed left legs having greater dorsal BMD and a tendency for higher medial BMD, this is suspected to be due to the handlers standing on the left side of the treadmill, which may have influenced the sheep’s posture and gait. This human influence has been documented previously in treadmill-exercised animals [21].

While only tendencies for treatment differences, these data exemplify the suspected asymmetric adaptations of bone to circular exercise due to diameter and speed. More conclusive differences in these parameters may be able to be detected with even faster speeds or a greater number of animals. These data represent the expected physiological differences in response to exercise and clarify where future research efforts should be focused to determine the effects of different training regimens on bone characteristics.

Though no significant differences were found regarding treatment, leg, or treatment by leg for PGE_2_, nor effects of treatment on DNA or GAG as markers of joint health, it cannot be ruled out that circular exercise may have detrimental effects on joints. In this study, sheep were exercised at relatively slow speeds. Thus, at these circle diameters, it is possible that the lean angle of the animals did not reach a degree that would elucidate clear treatment or leg differences, such as that of cantering or galloping in a horse. Similarly, for the sheep exercised in the treadmill treatment groups, it was not expected that a speed of 2.0 m/s would result in overloading of the joints and subsequent damage. However, evidence from bone density analysis, fracture force, and serum markers of bone formation and resorption indicates that exercise regimens did elicit differences in loading patterns between legs or treatment groups. This is important to keep in mind, as what is beneficial for bone may not necessarily be beneficial for joints. It is known that damage to supporting joint structures, joint capsules, or the articular cartilage itself may result from overuse or overloading and can subsequently result in osteochondral damage, synovitis, and development of OA [7,26,45,46]. Even during normal, symmetric loading of the limbs, loads placed on cartilage are not uniform across the joint surface [47,48]. Thus, as loading of the limb increases, focal points on the joint surface may experience excessive force even if below the strain threshold of the bone.

In the case of circular exercise specifically, the risk of joint damage may be increased further due to the resultant asymmetries in movement and loading as evidenced by the results of this study and others. Asymmetric joint changes have been documented previously in response to circular exercise by Logan et al. in 2022, with GAG concentrations of cartilage from the proximal surface of the MCIII and IV being greater for the outside leg than the inside leg of calves exercised on a small diameter circle [21]. However, conclusive evidence regarding the effects of circular exercise on joint health and characteristics has not yet been found in the species in which it has been studied. A limitation to this study is that data for DNA and GAG analysis were required to be grouped by leg, as preliminary statistical analysis found leg differences across all treatment groups. This is suspected to be due to samples from the right and left legs each being collected by different individuals resulting in an erroneous statistical finding due to the sampling procedure. Thus, treatment differences were analyzed for right and left legs independently. Further, it is possible that differences were not found due to sampling a single joint location, and changes could have occurred at different anatomical sites. However, these data still provided valuable insight into the effects on joint health under these varying exercise protocols and provide direction for future investigations.

The effects of circular exercise on bone and joint health may be able to be further determined by future studies in which a greater lean angle is induced either by faster speeds, smaller circle diameters, or both. Additionally, data collection on stride rate and gait kinematics that allows for the calculation of strain rate would be beneficial in determining the specific loads placed on the distal forelimb in addition to a recording system to determine specific lean angles across treatments. Future considerations should also include how these effects in horses are influenced by the presence of a rider, the frequency and intensity of circular exercise, and the gait at which these circles are performed. It is also warranted to compare multi-directional training sessions to single-direction training sessions to investigate how a change in direction may influence adaptations of the musculoskeletal system. However, in the current study, even relatively slow speeds and moderate circle diameters resulted in tendencies for changes to the bone regarding BMD, EI, and fracture force, while treatment differences were seen regarding markers of bone formation and resorption. These results indicate that circular exercise is likely to result in asymmetric musculoskeletal adaptations, supporting previous work, and provides further evidence of the importance of speed to stimulate enhanced bone formation and strength.

## 5. Conclusions

Circular exercise is frequently used across many equestrian disciplines, but it may be at the detriment of joint health for the horse and result in asymmetric bone adaptations. Based on the results of this study, circular exercise influences musculoskeletal characteristics indicative of bone quality, likely through asymmetric loading. Fracture force of the MCIII and IV tended to be higher in the outside leg of the large fast exercise group. BMD tended to decrease on the outside leg of the large slow exercise groups and increased on the outside leg in the small slow exercise group relative to the inside leg. Analysis of serum biomarkers also indicated that greater exercise speeds result in greater bone formation activity and slower speeds result in greater bone resorption activity, further implicating the importance of speed, and thus strain, in bone metabolism and why this must be considered regarding circular exercise. Changes in loading may also influence joint health when exercise is performed at faster speeds or smaller circle diameters. Thus, the combination of speed and circle diameter is an important factor to consider when designing a training program as well as in the evaluation of common practices within a discipline. Further studies are needed to determine the full effects of circular exercise on bone and joint health, and how these effects may be influenced by the presence of a rider, gait, session intensity, or changes in direction. Despite the need for continued research in this area, the results from the present study suggest that circular exercise does indeed result in asymmetric physiological changes to the musculoskeletal system.

## Figures and Tables

**Table 1:** Exercise protocol for treatment groups based on speed and total distance completed per exercise session weekly.

	Week of Treatment					
	1	2	3	4	5	6–12
Slow Treatment Groups						
Time (min) at 1.3 m/s	5	10	15	20	25	30
Distance (m) at 1.3 m/s	390	780	1170	1560	1950	2340
**Total Distance (m)**	390	780	1170	1560	1950	2340
Fast Treatment Groups						
Time (min) at 1.3 m/s	4	8	12	16	20	24
Distance (m) at 1.3 m/s	312	624	936	1248	1560	1872
Time (min) at 2.0 m/s	0.7	1.3	2.0	2.6	3.3	3.9
Distance (m) at 2.0 m/s	78	156	234	312	390	468
**Total Distance (m)**	390	780	1170	1560	1950	2340

**Table 2:** Bone mineral density (BMD, mg mineral/cm^3^) of the dorsal, palmar, medial, and lateral aspects at the midpoint of the fused third and fourth metacarpal (MCIII and IV) of juvenile sheep by leg and treatment group.

Treatment	Leg ^1^	Dorsal	Palmar	Medial	Lateral
Control	Right	1316	988	1315	1319
	Left	1303	1015	1311	1271
Straight Slow	Right	1320	1076	1331	1315
	Left	1296	1036	1331	1320
Small Slow	Right (inside)	1304	1049	1324	1318
	Left (outside)	1333 †	1052	1334	1333
Large Slow	Right (inside)	1291	992	1321	1298
	Left (outside)	1285	975	1272 *	1277
Straight Fast	Right	1298	1009	1303	1339
	Left	1347 *	1036	1347 *	1332
Small Fast	Right (inside)	1314	1023	1310	1324
	Left (outside)	1304	1037	1324	1333
Large Fast	Right (inside)	1318	1010	1316	1308
	Left (outside)	1301	994	1322	1335
	**SEM**	30.5	46.0	25.5	24.2

^1^ For circular exercise groups, the left and right legs were always the outside and inside legs, respectively. Exercise was conducted tracking right (clockwise). * Indicates significant difference between right and left legs within a treatment group at *p* ≤ 0.05. † Indicates trend for difference between right and left legs within a treatment group at *p* ≤ 0.1.

**Table 3:** Fracture force (ultimate strength, N) of the fused third and fourth metacarpal (MCIII and IV) of juvenile sheep by treatment group and leg.

Treatment	Leg ^1^	Fracture Force (N)
Control	Right	2140
	Left	2180
Straight Slow	Right	2270
	Left	2360
Small Slow	Right (inside)	2550
	Left (outside)	2550
Large Slow	Right (inside)	2350
	Left (outside)	2280
Straight Fast	Right	2280
	Left	2250
Small Fast	Right (inside)	2430
	Left (outside)	2490
Large Fast	Right (inside)	2220
	Left (outside)	2310 †
	**SEM**	114

^1^ For circular exercise groups, the left and right legs were always the outside and inside legs, respectively. Exercise was conducted tracking right (clockwise). † Indicates a trend at *p* ≤ 0.1 for inside and outside legs to differ within the large fast treatment group.

**Table 4:** Serum osteocalcin (OCN) levels in ng/mL measured biweekly across the 12 wk study.

		Day						
		0	14	28	42	56	70	82
Treatment	Overall Average ^1^	125 ^w^	102 ^x^	94 ^xy^	79 ^z^	84 ^yz^	91 ^xy^	84 ^yz^
Control	96 ^ab^	131	98	91	81	79	98	92
Straight Slow	85 ^b^	117	97	77	74	72	84	76
Small Slow	91 ^b^	115	101	87	79	90	87	81
Large Slow	86 ^b^	123	88	94	66	72	84	78
Straight Fast	104 ^a^	145	108	104	90	93	95	93
Small Fast	104 ^a^	135	111	115	84	95	96	92
Large Fast	92 ^b^	111	108	84	79	88	95	79
**SEM**	4	4	4	4	4	4	4	4

^1^ Overall serum OCN concentrations for each treatment (column values) and day (row values). ^a,b^ Treatments lacking a common superscript differ at *p* ≤ 0.05. ^w,x,y,z^ Days lacking a common superscript differ at *p* ≤ 0.05.

**Table 5:** Serum crosslinked C-telopeptides of type-I collagen (CTX-1) levels in ng/mL measured biweekly across the 12 wk study period by treatment and day.

		Day						
		0	14	28	42	56	70	82
Treatment	Overall Average ^1^	0.60 ^yz^	0.57 ^z^	0.55 ^z^	0.65 ^xy^	0.60 ^yz^	0.59 ^yz^	0.71 ^x^
Control	0.59 ^bc^	0.66	0.53	0.53	0.66	0.58	0.53	0.66
Straight Slow	0.60 ^b^	0.60	0.61	0.60	0.65	0.52	0.54	0.71
Small Slow	0.71 ^a^	0.68	0.57	0.57	0.72	0.79	0.72	0.92
Large Slow	0.67 ^a^	0.72	0.58	0.61	0.67	0.77	0.60	0.75
Straight Fast	0.53 ^c^	0.49	0.53	0.49	0.60	0.45	0.55	0.61
Small Fast	0.60 ^bc^	0.54	0.65	0.52	0.66	0.55	0.58	0.69
Large Fast	0.56 ^bc^	0.53	0.50	0.52	0.57	0.54	0.59	0.64
**SEM**	0.02	0.02	0.02	0.02	0.02	0.02	0.02	0.02

^1^ Overall serum CTX-1 concentrations for each treatment (column values) and day (row values). ^a,b,c^ Treatments lacking a common superscript differ at *p* ≤ 0.05. ^x,y,z^ Days lacking a common superscript differ at *p* ≤ 0.05.

**Table 6:** Ratio of osteocalcin (OCN) to crosslinked C-telopeptides of type-I collagen (CTX-1) measured biweekly across the 12 wk study by day and treatment.

		Day						
		0	14	28	42	56	70	82
Treatment	Overall Average ^1^	216 ^w^	189 ^x^	179 ^x^	126 ^z^	152 ^y^	166 ^xy^	123 ^z^
Control	167 ^bc^	200	196	177	127	138	190	144
Straight Slow	148 ^cd^	200	182	132	118	140	158	109
Small Slow	141 ^d^	175	181	160	117	124	133	93
Large Slow	132 ^d^	171	158	156	99	95	144	105
Straight Fast	199 ^a^	297	202	219	148	210	176	139
Small Fast	187 ^ab^	252	175	238	134	186	180	146
Large Fast	177 ^ab^	220	229	173	138	169	185	126
**SEM**	8	8	8	8	8	8	8	8

^1^ Overall serum ratio of OCN to CTX-1 for each treatment (column values) and day (row values). ^a,b,c,d^ Treatments lacking a common superscript differ at *p* ≤ 0.05. ^w,x,y,z^ Days lacking a common superscript differ at *p* ≤ 0.05.

## Data Availability

The raw data supporting the conclusions of this article will be made available by the authors on request.
